# Advances in Machine Learning Models for Predicting Enzyme Kinetic Parameters

**DOI:** 10.1021/acs.jcim.5c02428

**Published:** 2025-12-17

**Authors:** Ali Malli, Denys Vasyutyn, Jin Ryoun Kim

**Affiliations:** † Department of Chemical and Biomolecular Engineering, 34242 New York University, 6 MetroTech Center, Brooklyn, New York 11201, United States

**Keywords:** artificial intelligence, catalytic activity, enzyme engineering, enzyme evolution, enzyme mining, kinetic parameters, machine learning

## Abstract

Enzyme kinetic parameters, including *k*_cat_, *K*_m_, *k*_cat_/*K*_m_, and *K*_i_, are critical for guiding applications in enzyme engineering, metabolic modeling, and synthetic biology by providing quantitative information on enzyme activity under various conditions. Experimental determination of these parameters is often costly and time-consuming. Moreover, traditional computational methods are not well-suited to estimating these parameters. This motivated the development of machine learning (ML) models for *in silico* predictions. Here, we review recent advances in ML-based prediction of enzyme kinetic parameters by highlighting global models trained on diverse enzyme classes and local models catered toward specific enzyme families. These models have been applied in myriads of applications including predicting mutation effects, accelerating enzyme mining, and parametrizing genome-scale metabolic models. While data scarcity remains the main limitation for these models, we outline emerging opportunities such as high-throughput data generation and semisupervised learning as means to overcome this issue. In summary, this Review provides a roadmap for leveraging ML to enhance the performance, robustness, and scope of enzyme kinetic parameter prediction, leading to the accurate annotation of protein sequences for target functions.

## Graphical abstract

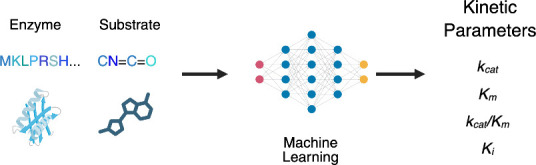

## 1 Introduction

Enzymes are natural catalysts that selectively and efficiently transform chemical compounds by accelerating chemical reactions.^1,2^ Advances in recombinant DNA and gene cloning technologies have facilitated the scalable expression of enzymes in microbial hosts, making them attractive candidates for the synthesis of value-added chemicals.^3,4^ In the context of the transition toward a circular economy, these bioprocesses provide sustainable pathways for the production of materials and energy.^5^ Nevertheless, the effectiveness of these processes depends on a set of kinetic parameters that govern the rate and efficiency at which enzymes catalyze substrates into products. Hence, optimizing enzymatic systems for industrial applications becomes essential as it demands meticulous engineering and screening to achieve catalytic activity and stability compatible with process conditions.^6,7^

Though enzymes are usually built from only 20 amino acids, they exhibit a great diversity. For instance, there are 20^100^ combinatorial possibilities for a typical peptide chain of 100 residues, which exceeds the number of known particles in the universe.^8^ However, it is estimated that only 1 of 10^77^ of these sequences folds into a stable and functional structure.^8,9^ Traditionally, navigating these sequences is facilitated by experimental approaches such as directed evolution to find enhanced mutants of known enzymes^10,11^ or metagenomic mining to discover new enzymes.^12^ While these strategies narrow the search space, their dependence on costly, time-consuming, and iterative assays poses an obstacle to scaling.^13,14^ This leaves a large portion of enzymes uncharacterized, given the vastness of the sequence space.^15,16^

Despite the limitations discussed above, the experimental efforts to search the sequence space resulted in a surge in the size of the available data. This permits the use of statistical methods to reveal hidden patterns and relationships. Hence, a promising alternative that has been gaining a lot of traction in recent years relies on advances in artificial intelligence to fit models to the available data and make *in silico* predictions regarding enzymatic properties.^17,18^ To this extent, machine learning (ML) and deep learning (DL) models have been successfully used for predicting enzyme structure,^19–21^ function,^22,23^ and fitness.^24–29^ The fitness landscape is composed of multiple features, such as activity, stability, and expression levels, that contribute to the overall performance.^30,31^ However, fitness scores do not directly quantify catalytic performance under reaction conditions.^32,33^ Also, computational tools to predict activity are still lacking compared to those developed for predicting stability and expression. Hence, a shift from generic fitness proxies to parameter-specific predictions represents a more interpretable approach for engineering enzymes with the desired catalytic properties.

When it comes to quantifying activity, ML models should make predictions for kinetic parameters, such as the turnover number (*k*_cat_), the Michaelis constant (*K*_m_), the catalytic efficiency (*k*_cat_/*K*_m_), and the inhibition constant (*K*_i_). Each parameter represents enzyme activity under different conditions, such as varying substrate concentrations and the presence or absence of inhibitors. Briefly, *k*_cat_ represents the maximal number of substrate molecules converted to products per active site of enzymes per unit time, while *K*_m_ is equivalent to the concentration of the substrate at which the enzyme functions at half of its maximal catalytic rate. The ratio of these two constants represents the efficiency of the enzyme in converting substrate to product, considering the enzyme’s speed and its affinity toward the substrate.^34^ Meanwhile, when an inhibitor is present, it is important to account for *K*_i_, which quantifies the strength of the inhibitor in blocking the activity of the enzyme.^35^ In some cases, *k*_cat_ and *K*_m_ can be theoretically determined from the activation free energy and substrate binding energy, respectively.^36^ However, in practice, estimating transition state energies at atomic accuracy is computationally challenging for most enzymes.^37^ Moreover, *K*_m_ does not always directly reflect substrate–enzyme binding affinity, as it can also be influenced by other kinetic steps.^34^ These challenges make it difficult to reliably estimate enzyme kinetic parameters directly from physical principles, highlighting the potential of ML methods trained on experimental data to bridge this gap.

Early work in this space applied ML algorithms to small curated data sets,^38,39^ but the field is rapidly evolving to cover large data sets of enzyme–substrate pairs (Figure 1). Fast and accurate predictions of these parameters can accelerate the discovery of efficient enzymes suitable for desired biochemical reactions by avoiding sequences with poor *k*_cat_/*K*_m_ or that suffer from strong product inhibition.^40^ Moreover, they can assist in genome-scale metabolic modeling^39^ and biosynthetic pathway prescreening by providing quantitative information on constituting reactions.^41^

**Figure 1:**
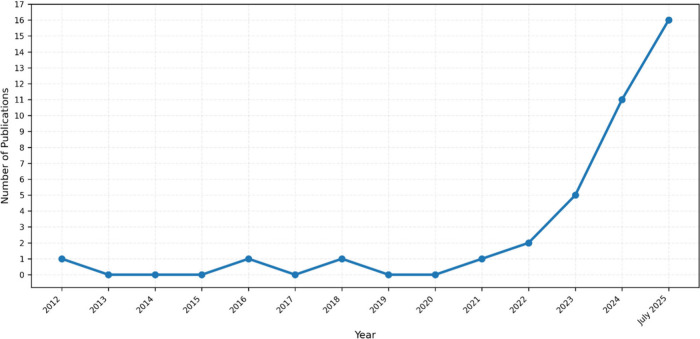
Trend in the number of ML and DL models published for enzyme kinetic parameter estimation. This illustrates the increasing attention that these models are receiving as a useful tool in enzyme engineering.

This Review aims to synthesize the current landscape of ML- and DL-based predictions of enzyme kinetic parameters. We begin by providing the reader with a background on ML basics in the context of enzyme kinetics (Section 2). Then, we highlight the common data sets available for kinetic parameter data (Section 3). Thereafter, we provide a roadmap of existing models summarizing their inputs, architecture, capabilities, and performance (Section 4). We also shed light on several applications in which these models have proven successful (Section 5) and discuss local models designed for specific enzymatic families (Section 6). Finally, we discuss the major challenges that these models face and propose directions for future research (Section 7).

## 2 Basic Elements of Machine Learning in Enzyme Kinetics

To gain a general view of the basic principles of ML in protein engineering, we refer the readers to a comprehensive review by Kouba and colleagues (2023)^17^ and Tables S1–S3 of the Supporting Information. Here, we provide a brief description of the tools and model architectures developed for enzyme kinetic parameters. ML models for enzyme kinetics prediction typically take two main inputs: the enzyme, represented by its amino acid sequence or 3D-structure, and the substrate, represented by chemical structure, simplified molecular input line entry system (SMILES) strings, or molecular fingerprints.^42^ These representations convert molecular information about enzymes and substrates to mathematically tractable formats while minimizing information loss (Figure 2a). Various neural network architectures excel at encoding each modality, but usually require relatively large data sets for training in order to achieve high performance.^42^

**Figure 2:**
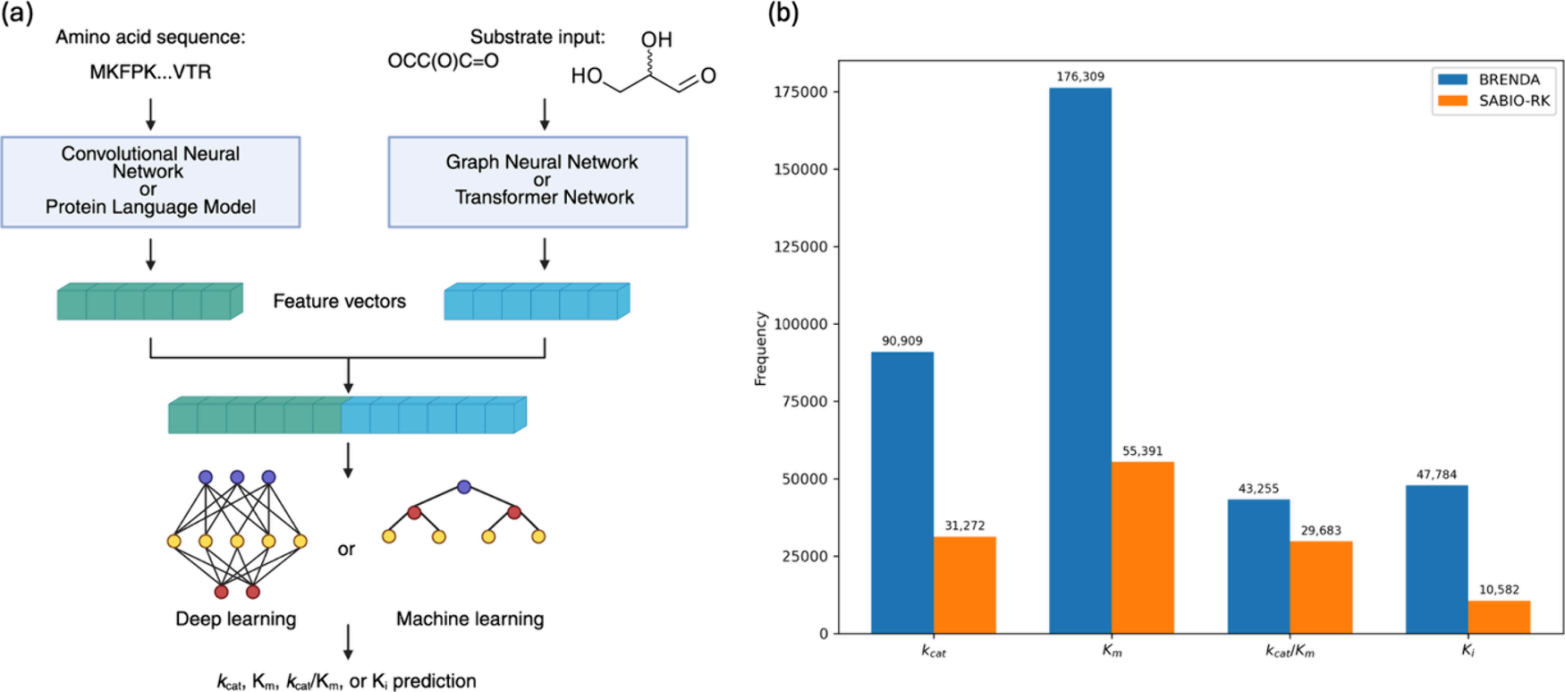
Overview of ML workflows for enzyme kinetic parameter prediction and enzyme kinetic data sets. (a) Schematic of typical model architectures and (b) number of available kinetic parameter data in major public databases as of July 2025.

A simple method for extracting enzyme sequence features is to use one-hot encoding, where each amino acid is represented as a binary vector with “1” indicating its presence and “0” indicating its absence at residue position *i*. However, this leads to sparse and high dimensional feature vectors compromising computational efficiency.^43^ Meanwhile, convolutional neural networks (CNNs) extract local motifs using sliding kernels to output *n*-gram frequencies or catalytic site patterns.^44^ The number of features from *n*-grams can grow extremely large, potentially becoming a high dimensional feature space as *n* increases. While CNNs are efficient for detecting short functional motifs, they fail to capture long-range dependencies, for example intramolecular interactions between amino acids that are distant in the primary sequence.^45^ This issue can be overcome using protein language models (pLMs). In natural language processing (NLP), language models treat sentences as sequences of words and learn their contextual relationships via a form of self-supervised learning called transformer networks.^46,47^ Similarly, enzyme sequences can be interpreted as sentences and tokenized using the individual amino acids of which they are comprised of. This parallelism opened the door to creating pLMs that are trained to predict masked amino acids in enzyme sequences.^48^ Ultimately, the outputs from pLMs, called embeddings, hold essential functional and structural information about the enzyme sequences, encompassing both short- and long-range relationships. To make predictions about enzyme kinetic parameters, enzyme sequences can be numerically encoded using pretrained pLMs such as Evolutionary Scale Modeling 2 (ESM2),^23^ ProtT5,^49^ and UniRep.^50^ These models are trained on millions of unlabeled protein sequences to learn biologically relevant features without requiring functional labels. These models generate a high-dimensional embedding for each amino-acid residue. However, since ML models usually require one vector per enzyme rather than one per residue, these embeddings are compressed into a fixed-length enzyme-level representation by applying pooling operations over the final transformer layer. On average, these features are considered high-dimensional, as the length of a pLM vector based on the last transformer layer is ∼1000–1300.

When structural data are used, a variety of information encoding strategies could be utilized. A simple method for capturing structural information would be to count the number of specific residues within the radius of a point of interest (e.g., active site).^51^ Alternatively, enzyme structures can be converted to residue–residue graphs, called contact maps, where the nodes represent amino acids and the edges depict spatial proximity and interactions.^52^ This information can be encoded by using graph convolutional networks (GCNs) or graph attention networks (GANs). GCNs aggregate information from neighboring residues in the graph and propagate local structural features across the enzyme. Meanwhile, GANs extend GCNs by introducing attention weights that allow the model to focus on important neighbors, such as catalytic residues and substrate binding sites, rather than treating all neighbors equally.

As for substrates, they can be encoded as fingerprints using various simple molecular descriptors that were historically used by the chemical community to describe small molecules like MACCS keys that tabulate the presence of substructures and functional groups into fixed-length vectors.^53^ To encode substrate structures as molecular graphs, GCNs and GANs are used to aggregate atom-level features such as partial charges and aromaticity and weight atom–atom connections attributed to chemically relevant interactions. Moreover, message passing neural networks (MPNNs) extend this by explicitly modeling iterative message exchanges between atoms to depict long-range effects in the molecule.^54^ Finally, akin to pLMs, transformer-based networks such as the SMILES transformer^55^ and ChemBERTa^56^ have been developed to tokenize SMILES strings into numerical features by learning substructure relationships and chemical grammar from large molecular data sets.

## 3 Databases

ML models used for complex tasks, such as predicting enzyme kinetic parameters, require large sets of training data to generalize well and generate reliable predictions. Such information is publicly available in large databases like BRaunschweig ENzyme DAtabase (BRENDA),^57^ System for the Analysis of BIOchemical Pathways - Reaction Kinetics (SABIO-RK),^58^ and UNIversal PROTein resource (UniProt).^59^

BRENDA is one of the most comprehensive repositories of experimental enzyme data extracted from more than 100,000 literature references.^57^ Besides kinetic parameters, the database contains information about the enzyme’s classification (EC) number, the source organism, and assay conditions, to name a few.^57^ Similarly, SABIO-RK is a database containing data about enzymatic reactions and their kinetic parameters manually retrieved from the literature.^58^ Whereas BRENDA focuses on enzymes and their kinetic parameters, SABIO-RK is centered around reactions and goes beyond kinetic constants to cover rate laws and experimental conditions.^57,58^ The number of enzyme kinetic parameters available in these databases is reported in Figure 2b. It is noteworthy to mention that there are fewer *k*_cat_/*K*_m_ and *K*_i_ entries in these databases compared to *k*_cat_ and *K*_m_.

As for UniProt, it is the largest database for proteins containing information about protein sequences, structures, and functions, as well as their functional parameters.^59^ Enzyme kinetic data in UniProt is scarce, with only around 1% of the enzymes listed in UniProt having an experimentally determined *k*_cat_ value.^59–61^ However, its importance lies in the UniProt IDs that are usually used as anchors to align kinetic records from BRENDA and SABIO-RK with their corresponding sequences. For example, Krishnan and colleagues (2025) proposed a structure-oriented kinetic database (SKiD) that integrates *k*_cat_ and *K*_m_ with their corresponding 3D-structure data for 13,654 enzyme–substrate pairs spanning six enzyme classes.^62^ In this database, kinetic data were retrieved from BRENDA, and the sequences were mapped to their Protein Data Bank (PDB) structures via UniProt IDs. After identifying the catalytic and binding sites for the substrates, docking energies were calculated and stored in the database using GNINA.^63^ A similar but smaller database of 1050 enzyme–substrate pairs called IntEnzyDB also exists.^64^ Moreover, Boorla & Maranas (2025) compiled CatPred-DB, a comprehensive data set of *k*_cat_, *K*_m_, and *K*_i_ values that integrates the UniProt IDs to map entries from BRENDA and SABIO-RK to their corresponding amino acid sequence identifiers and predicted 3D structures in the AlphaFold-2.0 database.^19,40^ CatPred-DB spans 23197 *k*_cat_, 41174 *K*_m_, and 11929 *K*_i_ entries expanding the enzyme sequence space by up to 60% compared to other ML kinetic data sets.^40^

In contrast to the discussed databases that compile experimentally measured values, GotEnzyme^65^ and GotEnzyme2 ^66^ represent a new class of predicted kinetic parameters repositories. The original GotEnzyme database provides *k*_cat_ values for over 25.7 million enzyme-compound pairs across 8099 organisms predicted from the pretrained DLKcat model (see Section 4.1.1).^65,67^ GotEnzyme2 extends this to a broader set of parameters, including *K*_m_ and *k*_cat_/*K*_m_, for 59.6 million entries by retraining a suite of ML models (see Section 4) on a curated set of experimental data and utilizing the best-performing strategy to make predictions for uncharacterized enzyme–substrate combinations.^66^ It is important to note that most of the kinetic constants in these databases lack experimental validation as the values are derived from ML regression models whose predictive performance on held-out experimental measurements reaches *R* ^2^ ≈ 0.5–0.7. Consequently, although GotEnzyme and GotEnzyme2 greatly expand the characterized enzymatic sequence space, they should not be treated as ground-truth kinetic data, as the propagation of predicted error can be misleading. Instead, these resources can complement, rather than replace, experimentally curated databases in hypothesis generation and preliminary screening in enzymatic applications.

One caveat regarding the public data sets is that their entries are nonstandardized, where the data are collected under a myriad of experimental conditions. Moreover, many entries have a large portion of missing metadata, such as temperature, pH, and substrate concentration. This heterogeneity complicates the process of detecting anomalies and outliers, especially when the sequence-substrate pair is associated with multiple reported values of kinetic parameters. To overcome this variability, some studies resort to taking the maximum reported value^67^ or computing the geometric mean of the available entries as a representative value.^68^ While these approaches help with reducing noise, they might overlook biologically meaningful variation and hence highlight the need for more structured and standardized data sets. Moreover, it has been reported that up to 20% of the entries in BRENDA were inconsistent with the results reported in their published references,^69^ likely due to human errors and erroneous replacements of units. When it comes to mapping substrate names to their respective SMILES notations, one or more of chemical and biological information databases, such as PubChem,^70^ KEGG,^71^ or ChEBI,^72^ are used. However, the same compounds can have nonidentical common names under different entries, resulting in inaccurate SMILES mapping and highlighting the need for a systematic substrate information retrieval pipeline.

To that extent, recent initiatives to improve the findability, accessibility, interoperability, and reusability (FAIR) of data have been proposed.^73^ The Beilstein-Institut proposed a set of guidelines called STandards for Reporting ENzymology DAta (STRENDA) to report the results of enzyme-related measurements, ranging from specifying reaction conditions to linking experimental information with the selected kinetic model and estimated kinetic parameters.^74^ These standards are tailored toward enzymes used in synthesis, ensuring that the reported data covers the catalytic parameters of the enzymes which remains optional in general protein databases.^75^ While these standards are increasingly being adopted in databases like BRENDA and SABIO-RK, the extent of their usage remains limited and is at the discretion of researchers. To facilitate this transition, validation tools such as EnzymeML^76^ and STRENDA DB^77^ have been created to automatically check and ensure that enzymology data are complete and valid before being published in a journal or database.

Lastly, it is important to note that most of the kinetic entries in the aforementioned databases are derived from *in vitro* measurements. As enzyme behavior *in vivo* significantly differs due to molecular crowding,^78^ protein–protein interactions,^79^ and post-translational regulations,^80^ metabolic models trained on *in vitro*-based predictions may not fully capture physiological kinetic properties. Hence, this limitation should be considered when using these databases to train models applied to *in vivo* systems.

## 4 Global Models

Most ML models developed for enzyme kinetic parameter prediction are inherently predictive in nature. They are designed to quantify catalytic properties within existing biochemical contexts rather than generate *de novo* enzymes, though generative frameworks are beginning to emerge as complementary tools. Within this predictive landscape, ML models can broadly be categorized as either global or local depending on the scope of their training data and intended applicability. Global models are trained on large data sets that span multiple enzyme families, classes, and organisms. In theory, this enables them to generalize across a wide diversity of sequences and reactions. Their strength lies in capturing extensive trends in sequence-function relationships and being applied to distantly related enzymes. In contrast, local models are tailored to a narrower sequence space, focusing on a single enzyme and its variants or a family of closely related enzymes. By leveraging high-quality data in this restricted domain, local models have the potential to capture fine-grained interactions, albeit at the cost of generalizability. In this section, we highlight the advances in global models to predict *k*_cat_, *K*_m_, *k*_cat_/*K*_m_, and *K*_i_, summarizing details about the models and their performance in Tables 1, 2, 3, and 4 respectively. The summary of the *k*_cat_ and *K*_m_ models not discussed in detail is provided in Tables S4 and S5.

**Table 1:** Characteristics and Performance of ML Models for Predicting *k*_cat_

model name	data set size	model architecture	enzyme sequence representation	substrate representation	other features	performance^a^
*k*_cat_ in *E. coli* ^39^ (Section 4.1.1)	215	random forest			enzyme structure, network interactions, biochemistry, assay conditions	*R* ^2^ = 0.34
DLKcat^67^ (Section 4.1.1)	16838	neural network	n-gram (CNN)	molecular graph (GNN)		*R* ^2^ = 0.44
TurNuP^68^ (Section 4.1.2)	4271	gradient boosting	ESP^84^		numerical reaction fingerprint	*R* ^2^ = 0.44^†^
CataPro^83^ (Section 4.1.2)	27658	neural network	ProtT5-XL	MolT5 and MACCS keys		*r* = 0.48^†^
UniKP^85^ (Section 4.1.3)	16838	ExtraTrees	ProtT5-XL-UniRef50	pretrained SMILES transformer		*R* ^2^ = 0.68
EF-UniKP^85^ (Section 4.1.4)	636	ExtraTrees	ProtT5-XL-UniRef50	pretrained SMILES transformer	pH	*R* ^2^ = 0.44
	572	ExtraTrees	ProtT5-XL-UniRef50	pretrained SMILES transformer	temp	*R* ^2^ = 0.38
DLTKcat^86^ (Section 4.1.4)	16249	neural network	n-gram (CNN)	molecular graph (GAN)	temp	*R* ^2^ = 0.66
PreTKcat^87^ (Section 4.1.4)	16249	ExtraTrees	ProtT5-XL-UniRef50	molecular graph (MolGNet)	temp	*R* ^2^ = 0.69
GELKcat^88^ (Section 4.1.5)	16838	neural network	n-gram (word2vec)	molecular graph (graph transformer)	enzyme structure (GCN)	*R* ^2^ = 0.56
SAKPE^89^ (Section 4.1.5)	31507	gradient boosting	ESM-C	Mole-BERT	catalytic and substrate binding sites from EasIFA	*R* ^2^ = 0.49^†^
OmniESI^90^ (Section 4.1.5)	23197	neural network	ESM-2	molecular graph (GCN)		*R* ^2^ = 0.41^†^
DeepEnzyme^61^ (Section 4.1.6)	11927	neural network	transformer	molecular fingerprint (GCN)		*R* ^2^ = 0.58
KcatNet^91^ (Section 4.1.6)	11757	neural network	ESM-2, ProtT5-XL-UniRef50	pretrained SMILES transformer	enzyme structure (GCN)	*R* ^2^ = 0.69
KinForm^92^ (Section 4.1.7)	35001	ExtraTrees	ESMC, ESM-2, ProtT5-XL-UniRef50	pretrained SMILES transformer		*R* ^2^ = 0.68^†^
CatPred^40^ (Section 4.1.8)	23197	neural network ensemble	ESM-2	D-MPNN	enzyme structure (E-GNN), sequence attention	*R* ^2^ = 0.61^†^
ENKIE^93^ (Section 4.1.8)	25648	Bayesian multilevel models			MetaNetX reaction identifier, MetaNetX substrate identifier, EC number, enzyme family	*R* ^2^ = 0.36^†^
CPI-Pred^94^ (Section 4.1.8)	11834	neural network	ESM-2	molecular fingerprint (MPNN)		*r* = 0.54^†^
RealKcat^95^ (Section 4.1.9)	30442	gradient boosting	ESM-2	ChemBERTa		accuracy = 0.89

a Metrics without a superscript are measured after random data split for training and testing. Metrics with the superscript “†” are measured after sequence-aware data splits for training and testing. *R* ^2^ is shown as a primary metric to compare the performance of models. When *R* ^2^ values were not reported, PCC or accuracy were used instead. The sections where each model appears were listed below their respective names.

**Table 2:** Characteristics and Performance of ML Models for Predicting *K*_m_

model name	data set size	model architecture	enzyme sequence representation	substrate representation	other features	performance^a^
Kroll_K_m_ ^115^	11675	gradient boosting	UniRep	molecular graph (GNN)	substrate molecular weight, substrate octanol–water partition coefficient	*R* ^2^ = 0.26^†^
MLAGO^116^	17151	random forest			EC number, substrate KEGG ID, organism KEGG ID	*R* ^2^ = 0.54
GraphKM^117^	19754	gradient boosting	ESM-2	molecular graph (GNN)		*r* = 0.59
CatPred^40^	41174	neural network ensemble	ESM-2	D-MPNN	structural features (E-GNN), sequence attention	*R* ^2^ = 0.53^†^
MPEK^118^	24585	neural network	ProtT5-XL	Mole-BERT	pH, temp, organism name	*R* ^2^ = 0.61
DLERK_m_ ^119^	10122	neural network	ESM-2	molecular fingerprint (RDKit)	reaction fingerprints (RXNFP)	*R* ^2^ = 0.59
UniKP^85^	11722	ExtraTrees	ProtT5-XL	pretrained SMILES transformer	temp, pH	*R* ^2^ = 0.53

a Metrics without a superscript are measured after random data split for training and testing; Metrics labeled with “†” are measured after sequence-aware data splits for training and testing. *R*_2_ is shown as a primary metric to compare the performance of models. When *R*_2_ values were not reported, PCC was used instead.

**Table 3:** Characteristics and Performance of ML Models for Predicting *k*_cat_/*K*_m_

model name	data set size	model architecture	enzyme sequence representation	substrate representation	other features	performance^a^
UniKP^85^	910	ExtraTrees	ProtT5-XL	pretrained SMILES transformer	temp, pH	*R* ^2^ = 0.65
EITLEM-Kinetics^120^	13388	neural network	ESM1v	MACCS Keys (RDKit)		*R* ^2^ = 0.68^†^
CataPro^83^	25831	neural network	ProtT5-XL	MolT5 and MACCS Keys		*r* = 0.41^†^
CPI-Pred^94^	8151	neural network	ESM-2	molecular fingerprint (MPNN)		*r* = 0.39^†^

a Metrics without a superscript are measured after random data split for training and testing; Metrics labeled with “†” are measured after sequence-aware data splits for training and testing. *R* ^2^ is shown as a primary metric to compare the performance of models. When *R* ^2^ values were not reported, PCC was used instead.

**Table 4:** Characteristics and Performance of ML Models for Predicting *K*_i_

model name	data set size	model architecture	enzyme sequence representation	substrate representation	other features	performance^a^
CatPred^40^	11929	neural network ensemble		D-MPNN	enzyme structure (E-GNN), sequence attention	*R* ^2^ = 0.45^†^
OmniESI^90^	11929	neural network	ESM-2	molecular graph (GCN)		*R* ^2^ = 0.54^†^
SAKPE^89^	10841	gradient boosting	ESM-C	Mole-BERT	catalytic and substrate binding sites from EasIFA	*R* ^2^ = 0.36^†^
CPI-Pred^94^	4341	neural network	ESM-2	molecular fingerprint (MPNN)		*r* = 0.66^†^

a Metrics without a superscript are measured after random data split for training and testing; Metrics labeled with “†” are measured after sequence-aware data splits for training and testing. *R* ^2^ is shown as a primary metric to compare the performance of models. When *R* ^2^ values were not reported, PCC was used instead.

### 4.1 Models for *k*_cat_ Prediction

#### 4.1.1 Early Approaches (Before 2023)

One of the first ML models to predict *k*_cat_ was developed by Heckmann and colleagues in 2018.^39^ They employed a random forest model to predict *in vitro* *k*_cat_ values for various enzyme reactions in *Escherichia coli* by leveraging diverse structural and biochemical features.^39^ With available *k*_cat_ values for enzymatic reactions in *E. coli* accounting for only 10% of all catalytic reactions,^81^ the training data consisted of 172 *k*_cat_ values for various endogenous *E. coli* enzymes and the model achieved a coefficient of determination *R* ^2^ = 0.34 on an independent test set.^39^ The authors found that the most important feature for predicting *k*_cat_ was the reaction flux calculated by parsimonious flux balance analyses.^39^ Nevertheless, the model’s applicability was limited as the input features used are available for only a small subset of enzymatic reactions from well-studied model organisms such as *E. coli*, *S. cerevisiae*, and *H. sapiens*.

In 2022, DLKcat, an organism-independent DL model that requires more accessible inputs, was developed by Li and colleagues.^67^ The model solely relies on the enzyme’s amino acid sequence and one of the reaction substrates to make its prediction across the space of all possible enzymatic reactions.^67^ The model predicted logarithmic *k*_cat_ values that were, on average, within 1 order of magnitude from the experimental values and achieved an *R* ^2^ = 0.44 on a random subset of the full data set used as a test set.^67,68^ However, the data set contains several entries for wild type enzymes and their mutants paired with the same substrate as well as the same enzyme sequence paired with different substrates. Since the data was randomly split without accounting for shared sequence identity between test and training sequences, the model suffered from data leakage between sets where 67.9% of the enzyme sequences in the test set were also present in the training set and 90% shared >99% sequence identity with those in the training set.^82^ Accordingly, DLKcat struggles to generalize to unseen sequences that share <60% identity with sequences in the training set: it achieves a negative *R* ^2^ value, implying that the prediction for a given enzyme–substrate pair is worse than using the average *k*_cat_ value of all reactions.^82^

#### 4.1.2 Dealing with Data Leakage

The data leakage observed in DLKcat can be avoided by splitting the data sets into training and test data in a way where enzymes with identical or highly similar amino acid sequences would not occur in both training and test sets (Figure 3a). One of the first models to do this was TurNuP, published by Kroll and colleagues in 2023.^68^ Its key innovation was combining numerical reaction fingerprints and pLM embeddings of enzyme sequences to predict *k*_cat_ for wild-type enzymes.^68^ Though trained on a smaller data set (*n* = 4271), TurNuP achieved better performance compared to DLKcat (*n* = 16838) on a test set with sequences dissimilar to training sequences (*R* ^2^ = 0.44), including an *R* ^2^ = 0.33 for sequences with <40% sequence identity to training sequences.^68^ Unlike the model published by Heckmann and colleagues (2018),^39^ reaction fluxes did not improve the performance of TurNuP.^39,68^ Similar to the sequence-aware split used in TurNuP, Wang and colleagues (2025) trained a model called CataPro on 10-fold cross-validation data sets created by clustering their *k*_cat_ entries such that no two sequences in the same cluster share more than 40% identity.^83^ The model achieved a Pearson’s correlation coefficient (PCC) *r* = 0.48 at low sequence identities between training and test sequences,^83^ further confirming that generalization to unseen enzymes critically depends on leakage-free splits.

**Figure 3:**
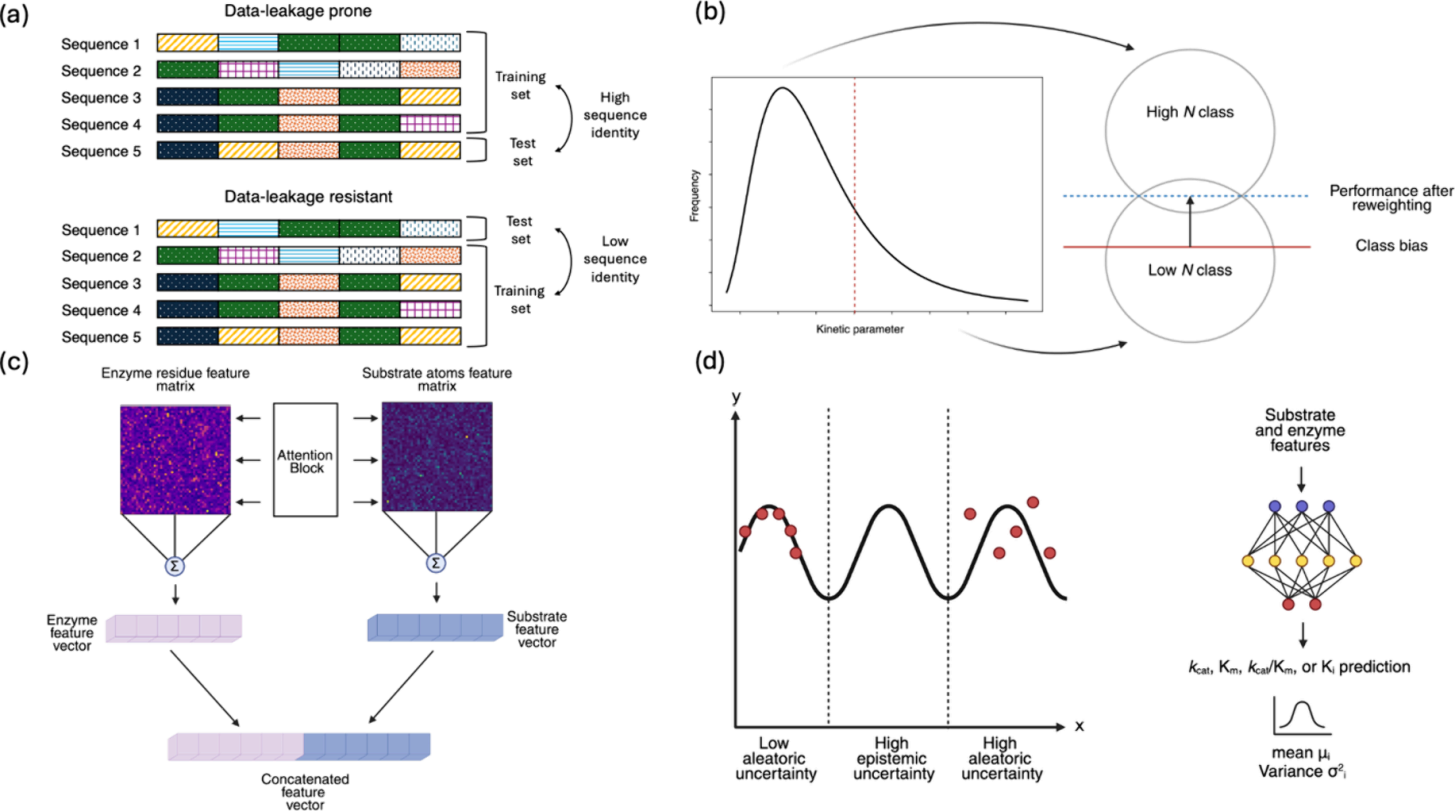
Strategies for improving predictive performance of ML enzyme kinetic parameter models. (a) Data splitting approaches to mitigate data leakage by controlling sequence identity between training and test sets, (b) addressing class imbalance in kinetic parameter distributions through reweighting schemes, (c) incorporation of interaction-aware features such as attention blocks to capture dependencies between enzymes and substrates, and (d) an uncertainty-aware modeling framework that distinguishes between aleatoric and epistemic uncertainty.

#### 4.1.3 Handling Data Imbalances

One limitation of TurNuP was its tendency to overestimate very low and underestimate very high *k*_cat_ values, which could be attributed to imbalances and noise in the data.^68^ The data imbalance issue was addressed by Yu and colleagues (2023) in their model UniKP using reweighting methods.^85^ The model achieved a decent performance owing to the use of an ensemble model which reduces variance, making the model more robust against the training data.^96^ However, upon analyzing the *k*_cat_ value distribution of the data set, the authors found that the absolute error is larger at both ends compared to the entries in the middle. Hence, they used class-balanced reweighting to improve the model’s ability to predict high values of *k*_cat_ by reducing the relative weight of the densely populated middle range of *k*_cat_ values. The reweighting was motivated by the argument that additional data points have diminishing returns to their corresponding class due to the overlap of information in the data set^97^ (Figure 3b). The RMSE of the reweighted model for high *k*_cat_ entries was 6.5% lower than that of the unweighted model.^85^

#### 4.1.4 Accounting for Environmental Factors

The noise in the data set could originate from the fact that the values of *k*_cat_ in the data set were measured under different experimental conditions such as temperature and pH. These environmental factors were not used as inputs to the previous models mostly due to their unavailability in existing data sets. Accordingly, Yu and colleagues (2023) created Revised UniKP, a retrained UniKP on a smaller data set containing either the temperature or pH at which the *k*_cat_ was measured, after retrieving the experimental conditions from UniProt.^85^ UniKP and Revised UniKP acted as base layers, whose outputs were fed to a linear regression layer to yield a final *k*_cat_ prediction at the pH or temperature of interest. This two-layer framework was called EF-UniKP and achieved an *R* ^2^ = 0.45 on the pH data set and an *R* ^2^ = 0.31 on the temperature data set when evaluated on a strict test set where either the enzyme or the substrate was not included in the training set.

While EF-UniKP did not assess the feature importance of environmental factors on their model, it provided a more realistic prediction of *k*_cat_ as it strongly depends on temperature.^98,99^ Another model that accounted for the temperature effects was DLTKcat.^86^ The model incorporated a bidirectional attention block to depict the interactions between substrate atoms and enzyme residues by computing attention weights in atom-to-residue and residue-to-atom directions as opposed to simply concatenating the vectors. Temperature values were added to the weighted vectors and fed into a set of dense layers to generate a *k*_cat_ prediction. Even though the model outperformed EF-UniKP achieving an *R* ^2^ = 0.66, it suffers from data leakage due to oversampling entries with low (<20 °C) and high (>40 °C) temperatures during data preparation.^87^ Since the test set was randomly chosen, some entries were susceptible to having identical matches seen during training, leading to the inflation of the performance metrics for DLTKcat. A more robust model to predict temperature-dependent *k*_cat_ is PreTKcat.^87^ PreTKcat uses an ExtraTrees ensemble model that achieved an *R* ^2^ = 0.69 on a 10-fold test set generated by a random split, a 2.98% improvement over UniKP.^87^ The authors reported that adding pH did not yield any significant improvement to the PreTKcat’s performance.^87^

#### 4.1.5 Using Interaction-Aware Features

Akin to DLTKcat, Du and colleagues (2025) argued that simply concatenating the substrate and sequence encoding vectors limits the ability to capture the complex feature interactions between the substrates and the enzymes^88^ (Figure 3c). Hence, they developed GELKcat that adopts a gate network that assigns weights to the enzyme and substrate encodings before fusing the vectors together and passing them to a fully connected neural network. Ablation studies showed that the gate network had a 3.92% improvement in *R* ^2^ compared to the model without it.^88^ Despite the gate network’s role, GELKcat did not outperform ML models that simply concatenated their feature vectors like UniKP and PreTKcat, likely due to their use of *n*-grams to represent enzyme sequences rather than pLM embeddings. In lieu of the attention blocks and gate networks, Qiu and colleagues (2025) opted to directly incorporate important sites for enzymes (e.g., catalytic and substrate binding sites) as features in their model SAKPE.^89^ This was performed using EasIFA, an algorithm that integrates pLMs with structural encodings to determine and assign weights to important residues in the enzyme sequence.^100^ Ablation studies showed that additional site features accounted for a 0.02 increase in the model’s *R* ^2^, and the model outperformed DLKcat and UniKP.^89^

More recently, OmniESI was introduced as a framework that predicts enzyme–substrate interactions using a conditional DL approach. Unlike the previous models that use concatenated static embeddings for their feature vectors, OmniESI relies on novel feature modulation strategies, bidirectional conditional feature modulation (BCFM) and catalysis-aware conditional feature modulation (CCFM), to refine the joint enzyme–substrate representation toward a catalysis-relevant latent space.^90^ Embeddings for enzyme sequences and substrates are fed to the BCFM where the enzyme acts as a condition for the substrate-side network, and the substrate acts as a condition for the enzyme-side network. Then, the modulated features enter the CCFM in parallel where their joint representation acts as a condition to reweight the features based on fine-grained biochemical dependencies. Ablation studies showed that these two modules increase OmniESI’s *R* ^2^ from 0.57 to 0.64.^90^

#### 4.1.6 Incorporating Enzyme Structural Features

To a large extent, enzyme function is governed by its 3D-structure,^101^ which none of the aforementioned models considered. Structure-based ML models have been widely used in other areas of protein science such as predicting protein function and ligand binding sites,^102–104^ yet few efforts have extended this approach to enzymatic kinetics. Wang and colleagues (2024) developed DeepEnzyme that, in addition to protein sequence and substrate structure, leverages protein structural features to make predictions for *k*_cat_.^61^ Since a large portion of kinetically characterized enzymes lack an experimentally determined 3D-structure, the authors used ColabFold^105^ to predict the structures of all enzymes in their data set. The model achieved an *R* ^2^ = 0.58 on its test set and maintained an impressive *R* ^2^ = 0.42 when the test sequences shared <50% sequence identity with the training sequences. The authors attribute this remarkable performance to the features learnt from the 3D-structures claiming that they closely correlate with function.^61^ Another model that accounts for structural features is KcatNet. It also incorporates an attention mechanism to capture interactions between the enzyme and the substrates in the feature encodings. The model outperformed UniKP by 18% on the same data set.^91^ Even though these models were tested for data leakage by considering sequence identity between test and training sequences, data leakage due to the structural similarity embedded in the contact maps was not considered. For example, two homologous enzymes can display highly conserved 3D-structures despite sharing low sequence similarity from diverging evolutionary paths.^106,107^

#### 4.1.7 Model Stability under High Dimensional pLMs

Some models may overfit the training data and appear deceptively strong due to the mismatch between the data size and the dimensions of the feature vectors. Here, we highlight strategies for improving pLM-based representations, either by selecting more informative layers or by reducing the dimensionality of the embeddings to mitigate overfitting. Alwer and Fleming (2025) built KinForm to address common issues in using pLMs to represent enzyme sequences.^92^ Since catalytic activity is often dictated by a handful of residues, uniform pooling of the pLM outputs might dilute relevant signals.^92^ Moreover, most of the models discussed thus far use the last transformer layer of the pLM which might not be the most informative for kinetic parameter predictions.^92^ Instead, the residue-level information was extracted from an intermediate layer of pLMs (e.g., ESMC and ESM-2) in KinForm. Moreover, the authors substituted uniform pooling with binding-site-weighted pooling to represent the more substrate binding relevant embeddings and concatenated them with the substrate representation vector. The substrate binding site weights were calculated separately using the Pseq2Sites model.^108^ To address the high dimensionality of the pLM vectors, principal component analysis (PCA) was used to reduce the vectors to 200–400 components which stabilized the model against overfitting.^92^ It is worth mentioning that KinForm outperformed UniKP, achieving *R* ^2^ roughly twice as high, especially in the low-similarity sequence bins.

#### 4.1.8 Uncertainty-Aware Models

All of the previous models are deterministic in nature, meaning that they use traditional regression approaches to output a single value *k*_cat_ prediction. Recently, Boorla and Maranas (2025) developed CatPred, a DL model that utilizes probabilistic regression to add a confidence metric to the *k*_cat_ predictions by estimating the associated uncertainties.^40^ They break down predictive uncertainties into aleatoric and epistemic uncertainty. The former is associated with any intrinsic noise in the training data while the latter results from the scarcity of training samples in a certain region of the latent space^40^ (Figure 3d). The authors imposed more lenient constraints during their data set preparation to end up with 23197 *k*_cat_ entries denoted as CatPred-DB. The model inputs were passed into a probabilistic regressor that outputs a Gaussian distribution of a *k*_cat_ predictions with a mean and a variance (Figure 3d). Since this variance only depicts the aleatoric uncertainty, the authors trained an ensemble of ten models under different initial weights where the average of the means is the final prediction and the variance of the means captures epistemic uncertainty.^40,109^ The model achieved an *R* ^2^ = 0.61 and *R* ^2^ = 0.39 on a held-out test set (which does not contain any enzyme substrate pair used for training) and an out of distribution test set (which does not contain any enzyme that shares >99% identity to a sequence used in training) respectively.^40^ Moreover, 76% of the predicted *k*_cat_ values fell within an order of magnitude from the actual value.^40^ Most of their predictions had a higher aleatoric uncertainty, which indicates high noise levels in the experimental data. Interestingly, unlike DeepEnzyme, structural features did not improve CatPred’s predictive performance. The authors attribute this finding to the fact that their pLM features encode not only sequence but also structural information.^40,110^ However, another model called DEKP showed through ablation studies that structural features enhanced their *R* ^2^ by 0.08, despite using a pLM to encode enzyme sequences.^111^ Overall, CatPred outperformed models like DLKcat and UniKP on the same out of the distribution set and showed a comparable performance to TurNuP under similar settings. This is likely due to the ensemble architecture of CatPred that provides robustness to the model’s predictions.

Building on probabilistic predictions, Gollub and colleagues (2024) introduced ENKIE, a Python package based on hierarchical Bayesian multilevel models (BMMs) to predict *k*_cat_ along with a calibrated confidence interval.^93^ Unlike the previous approaches that rely on enzyme sequence and substrate structures, ENKIE utilizes MetaNetX^112^ chemical identifiers for substrates and reactions, EC numbers for enzymes identifiers, and, optionally, the environmental conditions of the reaction.^93^ The model achieved an *R* ^2^ = 0.36 which is comparable to models developed by Heckmann and colleagues (2018)^39^ despite the simpler features. They also showed that the ENKIE estimates remain reliable even for unseen reactions.^93^ Lastly, Xu and colleagues (2025) integrated an error predictor that assesses the confidence level of *k*_cat_ predictions into their model CPI-Pred.^94^ The error predictor calculates Euclidean distances between test enzymes and structures against all entries in CPI-Pred’s training set using the embedding vectors and classifies the prediction as low or high confidence based on a threshold of 0.2 in absolute distance to a point in the training set. It achieved a 77% accuracy.^94^

#### 4.1.9 Reformulating the *k*_cat_ Prediction Task As a Classification Problem

Recently, RealKcat was introduced as a novel ML framework to predict *k*_cat_ using sequence and substrate embeddings.^95^ Unlike the previous models, the prediction task was treated as a classification problem clustering *k*_cat_ values into biologically meaningful order-of-magnitude bins. This approach aligns with industrial practices where the concern is largely about the order of magnitude of enzymatic activity rather than the exact numerical value.^113,114^ Due to the class imbalance caused by the lack of data on inefficient or inactive enzymes, the authors resort to a synthetic minority oversampling technique (SMOTE) in which the active sites of available sequences were substituted with alanine, creating a “zero activity” class. The model achieved an accuracy of 89% with 95% of the predictions falling within 1 order of magnitude from their true value.^95^

### 4.2 Models for *K*_m_ Prediction

Kroll and colleagues (2021) initiated the first endeavor to create an ML model to predict *K*_m_ on a large-scale data set.^115^ The model achieved an *R* ^2^ = 0.53 on the test set from BRENDA and an *R* ^2^ = 0.49 on an independent test set obtained from SABIO-RK. Moreover, it was robust against data leakage where the performance dropped minimally to *R* ^2^ = 0.45 when either the substrate or the enzyme in the test set was not present in the training data and *R* ^2^ = 0.26 when neither of them existed. Overall, the reported relative prediction error of the model was 4.1 on average, implying that the predictions deviate from the experimental values by around 4-folds.^115^

Meanwhile, Maeda and colleagues (2022) integrated ML into the traditional global optimization approach that optimizes kinetic model parameters to best fit experimental data.^116^ Since this traditional approach is time-consuming and often leads to biologically unreasonable solutions, they developed an ML-assisted global optimization approach (MLAGO) for *K*_m_ estimation.^116^ Similar to Kroll’s model, there was only a 4-fold difference between the measured *K*_m_ values and those predicted by MLAGO despite using simplified inputs (e.g., one-hot encodings). However, these predictions resulted in a high badness-of-fit to the experimental data (BOF > 0.02) when used for the carbon and nitrogen metabolism models. Accordingly, the predicted *K*_m_ values were utilized as a reference in the global optimization parameter estimation, and subsequently converged to values that fit the experimental data better (BOF < 0.02). In terms of limitations, MLAGO has low predictive power for extremely small or large *K*_m_ values, likely due to data imbalance.

More recently, He and Yan (2024) trained GraphKM on 19,754 sequences using a gradient boosting algorithm to predict *K*_m_.^117^ The model achieved an r = 0.59 outperforming the *K*_m_ model developed by Kroll and colleagues (2021) with an *r* = 0.23.^115^ Meanwhile, GraphKM’s performance against MLAGO was comparable on another independent test set.^117^ One downside of this model is that it relies on the maximum value of *K*_m_ reported in the literature for a given sequence rather than using the geometric mean of all observations. Hence, it runs the risk of consistently overestimating *K*_m_ and skewing the model’s predictions, especially if the maximum value reflected an outlier, error, or nonstandard assay conditions. Moreover, during data preparation, all sequences longer than 1000 amino acids were excluded, increasing the likelihood of the model generalizing poorly on long enzyme sequences. Finally, the data was split randomly even though 43% of their data set is mutant enzymes. Since the authors did not account for sequence identity between training and test sequences, the performance of GraphKM might be inflated and biased toward their specific data split.

Other models covered in Section 4.1 can also make predictions on *K*_m_ with the same feature representations and model architectures. Their performances are shown in Table 2 and S5 as well. For CatPred, it is worth mentioning that substrate features were solely able to explain 46.5% of the variance in the data when predicting *K*_m_ compared to 26% when predicting *k*_cat_.^40^ This is expected as *K*_m_ is related to binding affinity, which depends on how the substrate physically and chemically interacts with the enzyme. Meanwhile, *k*_cat_ is often governed more by the enzyme’s catalytic residues, transition states, and conformational flexibility rather than the substrate structure.

Wang and colleagues (2024) created MPEK, a multitask DL framework based on pLMs that predicts *k*_cat_ and *K*_m_ simultaneously, capturing the intrinsic connection between them due to its unique architecture.^118^ MPEK has a customized gate control framework composed of two expert layers (for *k*_cat_ and *K*_m_) and one shared expertise layer responsible for capturing this connection. The multitask learning in MPEK showed a 2.4% and 4.5% improvement in *R* ^2^ compared to single learning when predicting *k*_cat_ and *K*_m_, respectively.

Most of these models rely on enzyme–substrate pairs as inputs. However, akin to what was applied in TurNuP for *k*_cat_ prediction, Li and Wang (2025) considered the entire reaction, including both the substrates and products for *K*_m_ predictions, even though *K*_m_ is not directly related to the products. They trained DLERKm, a neural network with an attention channel on a database retrieved from SABIO-RK and UniProt that achieved *R* ^2^ = 0.59. Their model outperformed UniKP by 14.9%.^119^

### 4.3 Models for *k*_cat_/*K*_m_ Prediction

*k*_cat_/*K*_m_ serves as a fundamental kinetic parameter to measure the catalytic efficiency. However, ML models for *k*_cat_/*K*_m_ have been rare compared to those for *k*_cat_ and *K*_m_ (Table 3) mostly due to the lack of large available data sets for training. The first effort to integrate the task of *k*_cat_/*K*_m_ prediction to an ML model was in UniKP. Yu and colleagues (2023) retrieved a small data set of 910 entries to train and validate their model achieving an *R* ^2^ = 0.65 and an *r* = 0.81 with the experimental results.^85^ They compared the results to simply dividing the predicted *k*_cat_ and *K*_m_ values from their individual models (Tables 2 and 3) which achieved a low *r* = −0.02 with the experimental results.^85^ This is because taking the ratio of the independent predictions compounds the error associated with each model and ignores any correlation or codependency between both parameters. Hence, this stresses the need for a unified and task-specific model to predict *k*_cat_/*K*_m_. While UniKP demonstrates a good performance when predicting *k*_cat_/*K*_m_, it was not tested on any out-of-distribution sequences.

More recently, Shen and colleagues (2024) presented EITLEM-Kinetics, a novel approach for predicting *k*_cat_, *K*_m_, and *k*_cat_/*K*_m_ for both wild-type and mutant enzymes based on their sequences and one of the substrates involved in the reaction.^120^ They fine-tuned the pLM used to encode enzyme sequences by training attention blocks.^120^ This helped EITLEM-Kinetics learn which task-specific information is essential to be stored in a single protein representation rather than storing general protein information.^121^ Initially, the performance of the *k*_cat_/*K*_m_ neural network ensemble was significantly lower than that of the *k*_cat_ and *K*_m_ models. Accordingly, the authors enhanced the performance of the *k*_cat_/*K*_m_ model by leveraging transfer learning. To elaborate, since the DL models for *k*_cat_, *K*_m_, and *k*_cat_/*K*_m_ are related, it should be possible to transfer information and capabilities between the different prediction tasks. Moreover, training a model directly on *k*_cat_/*K*_m_ ignores the intricate relationships among the three parameters. Therefore, the authors iteratively readjusted the weight parameters of the *k*_cat_ and *K*_m_ networks based on the *k*_cat_/*K*_m_ model, retrained and fine-tuned the networks on their respective data sets, and then fed the new parameters into the *k*_cat_/*K*_m_ model again. After eight iterations, the performance of the *k*_cat_/*K*_m_ model increased from 0.61 to 0.83. Likewise, the performance of the *k*_cat_ and *K*_m_ models increased to 0.75 and 0.72 respectively. To ensure that the enhancement was not due to data leakage, the ensemble model was evaluated on a stricter data set excluding samples that overlap with the training set. Its performance for *k*_cat_/*K*_m_ still increased from 0.52 to 0.68. A likely reason iterative transfer learning improves model performance is that it provides a well-informed initial point for optimization. When the model is pretrained on a larger data set of enzymes, its parameters are guided toward a relevant region of the broad sequence space.^122^ Subsequent fine-tuning allows the model to gradually refine the parameters, making it less likely to be confined to a local minimum. Similarly, CataPro achieved an *r* = 0.41 when using transfer learning from their *k*_cat_ and *K*_m_ models to predict *k*_cat_/*K*_m_.^83^

### 4.4 Models for *K*_i_ Prediction

Relatively few ML models have been developed for *K*_i_ prediction for enzyme–inhibitor pairs whereas most ML research has focused on half-maximal inhibitory concentrations (IC50)^123^ and drug-target binding affinities (DTBA).^124^ Unlike IC50, which depends not only on inhibitor binding but also on downstream biological responses, *K*_i_ is a direct measure of enzyme–inhibitor binding affinity under different modes of action. By contrast, DTBA predictions typically address a wide set of drug–protein interactions, many of which are not enzymes, and thus offer limited insight into enzyme-specific inhibition. This distinction underscores the added value of ML approaches for *K*_i_ prediction. Four of the models discussed earlier have a *K*_i_ module: CatPred, SAKPE, CPI-Pred and OmniESI.

Similar to the *K*_m_ model, substrate features alone in CatPred could explain 52.5% of the variance.^40^ It is noteworthy to mention that the performance of CatPred deteriorated upon adding pLM features when evaluating an out of distribution *K*_i_ test set. This is a sign that the model is overfitting under high-dimensional pLM feature vectors due to the smaller size of the *K*_i_ data set. Accordingly, the authors replaced the pLM features with structural features that are lower in dimensions to achieve a good performance across both held out and out-of-distribution sets.^40^ When OmniESI was trained on the CatPred data set, it outperformed CatPred on all out-of-distribution test sets, surpassing its R^2^ by ∼11% at *a* < 40% sequence similarity cutoff.^90^ As for the *K*_i_ prediction task using SAKPE, the model achieved an *R* ^2^ = 0.77 on its test set.^89^ However, it shows signs of data leakage as the *R* ^2^ dropped to 0.36 and 0.29 when the test sequences shared <99% and <40% identity with the training sequences, respectively. In contrast, CPI-Pred was more robust as the PCC decreased from 0.76 on a randomly split data set to 0.61 when the test sequences shared <60% identity with the training sequences.^94^

## 5 Applications of Global Models

### 5.1 Predicting Effects of Mutations

The catalytic efficiency of natural enzymes often falls short of the requirements needed in industrial processes. Therefore, optimizing enzymatic activity becomes essential to reducing production and operation costs. Kinetic parameters of enzymatic reactions can be mapped to enzyme sequence by a conceptual landscape that is navigated through mutational walks.^31^ The effect of mutations on function is not additive,^125^ so the ability of a model to predict the impact of multiple mutations on enzymatic activity is a direct indicator of whether it understands residue–residue interactions and how they relate to the target kinetic parameters. DLKcat was able to capture the effects of amino acid substitutions on *k*_cat_ values by assessing their attention weights using a neural attention mechanism and achieved an *r* = 0.78 on the mutants in the test set.^67^ However, it has been proven that the model displayed inflated performance, as it recorded an *R* ^2^ = −0.18 when tested against sequences not present but still sharing more than 99% similarity with the training sequences.^82^ The authors of DLTKcat reported that the beneficial mutations for the enhancement of *k*_cat_ values were distributed near high peaks of attention weights from their GAN.^86^ However, since their model also suffers from data leakage, it is probable that the residues with high attention weights correspond to those of identical sequences in the training set. Furthermore, some high attention sites were observed at residues where no mutations were introduced, highlighting noise in the data.^111^

More robust models have demonstrated the ability to capture trends in kinetic parameter changes across enzyme mutants, underlining the varying degrees of success in predicting the effects of mutations. For instance, DeepEnzyme predicted a median *k*_cat_ value for highly active phosphate alkaline phosphatase mutants that was 15% higher than that of the low activity mutants.^61^ MPEK split their mutant data into wild-type-like, increased, and decreased *k*_cat_ or *K*_m_ categories. The model achieved PCC values for the prediction of mutant classes ranging between 0.8 and 0.9 for all categories across both parameters.^118^ Meanwhile, the authors of CataPro showed that their model is capable of ranking mutants based on the favorability of the mutations for a given substrate reaction (*r* > 0.7). However, it failed to capture the effect of these mutations quantitatively when a comprehensive evaluation was performed across the entire data set (*r* ≈ 0).^83^ Similarly, OmniESI offered a qualitative demonstration of their model’s strength against mutants by classifying single and double mutations in β-lactamase as beneficial or detrimental, achieving >85% accuracy against numerous substrates.^90^ EITLEM-Kinetics demonstrated a consistent predictive performance across most mutants with varying numbers of mutations, achieving an *R* ^2^ = 0.85 for up to six mutations on the *k*_cat_ data set. It also achieved an *R* ^2^ = 0.66 on mutants that showed more than a 10-fold increase in *k*_cat_. The model’s high performance in predicting the effect of mutations could be attributed to the fact that the features of each amino acid in EITLEM-Kinetics were represented individually rather than collectively by feature pooling. This, along with the attention networks used, makes the model more sensitive to mutations by capturing both enzyme-specific residue effects and broader sequence–substrate interactions.^120^

### 5.2 Enzyme Engineering and Mining

Engineering mutant enzymes with enhanced activity for specific biochemical reactions is a pivotal and typical objective in the fields of protein engineering and synthetic biology. However, identifying effective evolutionary pathways demands an exceptional understanding of reaction mechanisms to navigate the sequence space under biological and physical constraints, such as protein folding and expression. Likewise, resorting to laboratory directed evolution is costly, time-consuming, and labor-intensive and often yields only marginal success. For example, the directed evolution of a tyrosine ammonia lyase (TAL) library from *Rhodotorula glutinis* through the construction and screening of 4,800 mutants led to the identification of one variant with a *k*_cat_ of 142 s^–1^, only a slight improvement from the wild type with a *k*_cat_ of 114 s^–1^.^126^ To address the limited success of the experimental approach, the top 1000 homologues of the wild-type sequence were identified through a BLAST search and UniKP was used to predict their *k*_cat_ values for *in silico* enzyme mining. The top five predictions were experimentally validated, in which two sequences surpassed the wild type *k*_cat_ value by ∼4-fold.^85^ In parallel, UniKP was utilized to predict *k*_cat_/*K*_m_ values for all possible single-point variants of TAL from *R. glutinis* for *in silico* enzyme evolution. Ultimately, the authors identified and experimentally characterized two mutants that were up to 3.5-fold more efficient than the wild type. This demonstrates that UniKP learnt deep-level information about the enzymes, as sequences from the TAL mutant library shared <35% identity with the training sequences.^85^ Likewise, KcatNet was used for *in silico* evolution of α-glucosidase by screening all of its possible single-point mutants, the highest showing a 47% improvement in *k*_cat_ over the wild type.^91^ Lastly, CataPro was leveraged in the enzyme mining of more efficient carotenoid cleavage oxygenases. The authors started with the carotenoid cleavage oxygenase from *Caulobacter segnis* (CSO2) and identified 1500 homologues using BLAST.^127^ Experimental validation confirmed that the top hit among the CataPro predictions, *Sphingobium sp*. CSO (SsCSO), was 19.53 times more active than CSO2. Also, two rounds of *in silico* directed evolution on SsCSO using CataPro led to the identification of a double-point mutant that showed a 65-fold increase in activity compared to CSO2.^83^ However, it is critical to note that these models are more reliable for identifying relative trends, such as which mutants are likely to be more active, rather than for predicting the precise magnitude of mutation effects.

While these ML models have shown promise in predicting kinetic parameters for enzyme mutants, they are not specifically designed to suggest mutations that enhance the enzymatic activity. Yu and colleagues (2024) addressed this limitation by building a diffusion model that proposes multiple amino acid substitutions to optimize activity.^60^ They formulate this objective as an inverse folding task combined with a regressor-guided diffusion model denoted as *k*_cat_Diffuser.^60^ In other words, *k*_cat_Diffuser generates several enzyme sequences compatible with a given backbone structure while being guided by a sampling process favoring amino acid combinations that lead to higher *k*_cat_ values. It was trained on 15603 protein structures of BRENDA and CATH^128^ database entries generated using ESMFold.^23^ *k*_cat_Diffuser outperformed other diffusion models such as ProteinMPNN,^129^ PiFold,^130^ and GraDe-IF,^131^ generating mutants with an overall improvement of 0.21 in log *k*_cat_. For example, *k*_cat_Diffuser enhanced log *k*_cat_ for an undecaprenyl pyrophosphate synthetase activity by 0.486 while the aforementioned models generated less active mutants when prompted with the same task.^60^ The generated structures align well with the original enzyme structure, highlighting *k*_cat_Diffuser’s advantage in enhancing *k*_cat_ without compromising structural integrity.

Despite these successes, most of these models show limited interpolation of enzyme families that are not well represented in their training sets. For example, when tasked with predicting *k*_cat_/*K*_m_ for 260 β-glucosidases, UniKP merely predicted the same value for all enzyme sequences (*R* ^2^ = 0.05) while EITLEM-Kinetics and CataPro achieved *R* ^2^ values of 0.19 and 0.27, respectively.^132^ Similarly, when DLKcat was tasked with predicting *k*_cat_ values for 175 adenylate kinases, it achieved a Spearman’s coefficient of −0.09.^133^ These outcomes are well below the performance reported on the models’ respective test sets, and such inaccurate models can significantly hinder sequence exploration for enzyme mining and evolution, especially when targeting specific catalytic function. Accordingly, more realistic metrics are needed to assess whether ML models meaningfully support enzyme screening. Taken literally, regression metrics such as R^2^ and RMSE can lead to incorrect ranking of variants or failing to identify the true top performers. For applications such as enzyme mining or mutational scanning, metrics that evaluate enrichment in the top-performing region of the sequence space such as the enrichment factor (EF), precision@*k*, and recall@*k* offer a more actionable assessment,^134,135^ although they have primarily been used for evaluating functional fitness rather than predicting kinetic parameters. Notably, models with high *R* ^2^ can exhibit poor ranking fidelity or enrichment, rendering them less effective for enzyme engineering workflows. This is evident for models like UniKP where enzyme mining and evolution each resulted in only modest improvements (<3-fold) in *k*_cat_/*K*_m_.^85^

Lastly, evolving an enzyme for enhanced catalytic activity at a desired temperature is not feasible with current ML models, and experimental approaches involve timely and labor-intensive characterization steps. Recently, Erkanli and colleagues (2025) introduced a three-module ML framework that predicts the optimal temperature, *k*_cat_/*K*_m_, and the activity–temperature profile of β-glucosidases.^132^ By integrating both intrinsic sequence information and extrinsic temperature effects, the framework provides a powerful tool to traverse the sequence–temperature–activity landscape. Looking forward, similar models have the potential to accelerate enzyme evolution by guiding searches through vast sequence and temperature spaces simultaneously, uncovering novel functional mutants that operate under desired reaction conditions.

### 5.3 Genome-Scale Metabolic Modeling

Genome-scale metabolic models (GEMs) are mathematical representations of the complete set of metabolic reactions within an organism reconstructed from annotated genome sequences. They are advantageous in simulating the metabolic fluxes under different conditions, guiding metabolic engineering endeavors, and studying proteome allocation.^136,137^ Typically, GEMs are built on stoichiometric constraints derived from reaction networks and mass balance principles to estimate feasible reaction fluxes using methods such as flux balance analysis.^138^ However, their accuracy is limited by a key assumption: enzymes are treated as infinitely fast catalysts or enzymes are present in excess quantities.^139^ To address this, enzyme-constrained genome-scale metabolic models (ecGEMs) integrate enzyme capacity constraints, most commonly through *k*_cat_ values and enzyme abundances, thus linking the maximum achievable flux to the catalytic efficiency of the enzyme catalyzing the metabolic reaction.^140^ Despite their promise, ecGEMs remain hindered by incomplete or noisy kinetic data as many enzymes lack experimentally measured *k*_cat_ values.^39^ Moreover, available data often come from different organisms, assay conditions, and substrates, adding additional uncertainty. While ecGEMs have been developed for several well-studied organisms such as *E. coli*, only 10% of all enzymatic reactions have fully matched *k*_cat_ values in BRENDA, respectively.^67,141^ In addition, *in vivo* estimation of *k*_cat_ values from *in vitro* experimentation is difficult, especially due to their noncorrelative relationship.^69,81^ Without such data, ecGEMs usually adopt kinetic parameter values from similar substrates, reactions, or organisms, increasing the deviation of these models from experimental observations.

A way to mitigate the kinetic data bottleneck in ecGEMs construction is to use predicted *k*_cat_ from the ML models discussed to expand the coverage in genome-scale reconstructions. The model developed by Heckmann and colleagues (2018) aimed to parametrize GEMs for *E. coli* iML1515.^39^ The integration of ML-derived *k*_cat_ values instead of median-imputed ones from available data sets resulted in a substantial improvement (i.e., reducing the RMSE for their model by 34%^39^). One limitation of this approach is that the ML-predicted *k*_cat_ values are derived from *in vitro* rather than *in vivo* conditions. Similarly, DLKcat was trained on *in vitro* data, implying that the model would predict *in vitro* values as well.^67^ DLKcat was used to reconstruct the ecGEMs of 343 yeast/fungi species by predicting *k*_cat_ values for around three million enzyme–substrate pairs. To resolve the discrepancies between the *in vitro*-based predictions and *in vivo* values and ensure the biological relevancy of the predictions, the authors resorted to a Bayesian genome-scale modeling approach in which the DLKcat predictions serve as the mean and the model’s RMSE as the variance for prior *k*_cat_ distributions. Then, these values are updated iteratively based on experimentally measured phenotype data sets to produce posterior distributions that capture the uncertainty in the *k*_cat_ prediction. Overall, the DLKcat-based ecGEM’s RMSE was 30% lower than that of the original ecGCM.^67^ The authors of KcatNet parametrized the same ecGEM using their model and outperformed DLKcat in reducing the original model’s RMSE in 16 out of 22 carbon sources and oxygen conditions for four different yeast species.^91^

Meanwhile, DLTKcat was used to demonstrate how ML models can be used for temperature-sensitive metabolic modeling.^86^ The authors showed that DLTKcat predicted a decrease in the activity of catabolism in *Lactococcus lactis* MG1363 in response to a temperature increase, which is consistent with experimental observations. Moreover, it captured the increase in *k*_cat_ values for enzymes from *Streptococcus thermophilus* LMG18311 when temperature increased.^86^ However, when quantitatively estimating the growth rates, the model’s accuracy was extremely low, likely due to error propagation.

Beyond steady state ecGEMs, dynamic kinetic models are governed by full enzymatic rate laws, relying directly on all of the kinetic parameters and equilibrium constants. These models enable time-resolved simulations of metabolite dynamics and transient behavior that cannot be captured under a steady-state assumption. Kinetic models have been limited to small subsystems due to data scarcity, but recent advances have produced near-genome-scale kinetic reconstructions that integrate omics data, thermodynamic constraints, and parameter estimation frameworks to infer large sets of kinetic constants.^142^ Notable examples include large-scale kinetic models of *E. coli* metabolism,^143,144^ *S. cerevisiae* metabolism,^145,146^ and even human cancers.^147^ These kinetic models demonstrate the growing feasibility of large-scale parametrized ordinary differentia equation systems and highlight that ML-predicted *k*_cat_, *K*_m_, and *K*_i_ could play an increasingly central role in enabling genome-scale dynamic simulations.

To show how ML models can be used to fill in the gaps for missing *K*_m_ values in kinetic models, Kroll and colleagues (2021) leveraged their model to predict *K*_m_ for enzymes associated with 47 GEMs spanning *E. coli*, *S. cerevisiae*, *M. musculus*, and *H. sapiens*.^115^ While these organisms belong to different biological domains, their training data was dominated by bacterial data. Accordingly, they partitioned their test set by domain to illustrate that their model can make *K*_m_ predictions equally well for different domains, achieving *R* ^2^ values of 0.37, 0.51, and 0.56 for archaea, bacteria, and eukarya, respectively.^115^ Lastly, MLAGO was tasked with parametrizing the carbon and nitrogen metabolism models as the reactions’ corresponding *K*_m_ values were not included in the training set.^116^ While the *K*_m_ predictions achieved good accuracy (RMSE of 0.62 and 0.73 for carbon and nitrogen metabolisms, respectively), the ML-based metabolic models did not fit the experimental metabolic data with sufficient accuracy. When these *K*_m_ predictions were used as a reference for the MLAGO approach, the resulting metabolic models fit the experimental data well while still maintaining reasonable accuracy for the *K*_m_ predictions (RMSE of 0.79 and 0.57 for carbon and nitrogen metabolisms, respectively).^116^

## 6 Local Models

While many recent ML models aim to generalize parameter predictions across diverse enzyme families, one of the first models was developed for an enzyme family specific prediction task. Yan and colleagues (2012) aimed to predict *K*_m_ for β-glucosidases against their natural substrate, cellobiose, due to their importance in the biofuel industry.^148^ The model was constructed using a feedforward backpropagation neural network that takes the amino acid probability distributions as well as 11 properties from the AAIndex^149^ as an input. The network was trained on 24 β-glucosidase sequences and tested on another 12 achieving an *R* ^2^ = 0.67.^38^ However, this model suffers from overfitting as the choice of a neural network is inadequate with the extremely small size of the available data set.^150^ In 2016, Carlin and colleagues trained an ensemble of elastic net regressors on 100 mutants of the β-glucosidase from *Paenibacillus polymyxa*.^151^ They showed the ensemble approach was more robust than a single regressor, with PCC increases from 0.57 to 0.76 for *k*_cat_/*K*_m_, 0.43 to 0.6 for *k*_cat_, and 0.29 to 0.71 for 1/*K*_m_.^151^ The most important features were the hydrogen bonding energy of the substrate, polar contacts between the enzyme and substrate, and a minimal number of voids in enzymes for *k*_cat_/*K*_m,_ *k*_cat_, and *K*_m_, respectively. Several features were predictive for *k*_cat_/*K*_m_ but not for *k*_cat_ or *K*_m_; this is likely because there was no significant correlation between *k*_cat_ and *K*_m_ in the authors’ data set, making all the parameters independent. One of the limitations of this model was its bias toward low *k*_cat_/*K*_m_ values due to their over-representation in the training data set.

Local models with broader coverage have also been reported. Li and colleagues (2023) built a DL platform, DeepGH, for catalytic activity of glycoside hydrolases and applied this model to identify mutants with enhanced activity.^152^ DeepGH was trained on 64057 sequences retrieved from the CAZy database,^153^ spanning 119 glycoside hydrolase families and sharing at most 65% sequence similarity between training and test sets to avoid data leakage.^152^ The model takes a feature matrix containing information about the positions of the amino acids and their catalytic importance as an input. DeepGH was applied to chitosanase CHIS1754, identifying nine residues as target sites for mutations that could potentially enhance its activity. Experimental validation showed that eight of the nine single-point mutants were more active than the wild type. They also created the CHIS1754-MUT7 variant which includes seven of the nine suggested mutations from DeepGH, exhibiting a *k*_cat_/*K*_m_ that is 24-fold higher than that of the wild type.^152^

Shifting focus to other enzyme families, Muir and colleagues (2024) created a model focusing on adenylate kinases (ADK).^133^ Leveraging a high-throughput microfluidic platform, they measured *k*_cat_, *K*_m_, and *k*_cat_/*K*_m_ for 193 ADK orthologs and demonstrated that the ADK functional landscape is rugged and multipeaked, with the type of LID-domain – one of three domains of ADK – rather than phylogeny or reaction temperature playing the most critical role.^133^ Moreover, they fed ∼5000 ADK sequences to ESM-2 and observed that the outputs could be clustered by the LID-domain type as well, albeit not by activity. Taking advantage of pLM’s ability to capture high-level structural organization, the authors trained a random forest regressor on the ESM-2 embeddings of ADK sequences. This model achieved a Spearman’s rank correlation coefficient *r* = 0.44 on *k*_cat_ compared to *r* = −0.09 when they used DLKcat for the same test set. Furthermore, a support vector regressor was trained for *K*_m_ achieving *r* = 0.49.^133^ Despite using fewer sequences, this local model outperformed DL models trained on large data sets, underscoring the value of high-quality kinetic measurements within relatively narrow sequence spaces for building robust family specific predictors. It also highlights the limitations of the global models when trained on noisy and heterogeneous public data sets like BRENDA.

Meanwhile, Ao and colleagues (2023) incorporated structural features for their transaminase-specific model.^154^ They trained a gradient boosting regression tree on data-specific activity data from *E. coli* transaminase and 40 of its variants against 28 different substrates.^154^ They claimed that the enzyme sequences and structures contained a lot of information that was not directly relevant to the activity prediction task, depressing the performance of the ML model. Hence, they used only the electronic and steric properties of 14 amino acids at the mutation sites and 8 substrate descriptors as features. Their model achieved an *R* ^2^ = 0.80 for activity prediction, with three amino acids showing the highest feature importance. Using this model, more active mutants were identified with up to 3.5 fold increase in activity compared to previously identified variants.^154^

## 7 Limitations and Future Directions

### 7.1 The Dilemma of Limited Data

Despite the value of the existing databases, the sparsity and uneven distribution of kinetic measurements across enzyme classes remain a major challenge, as evidenced by our analysis of BRENDA (Figure 4). The majority of the characterized *k*_cat_ and *K*_m_ values belong to a small subset of hydrolases, oxidoreductases, and transferases against their natural substrates (Figure 4a). As a result, most ML models show strong performance in interpolating to these families but struggle to generalize to underrepresented enzyme families and non-natural substrates. Moreover, the main issue with collecting kinetic data sets from published literature is the inherent bias toward parameters with intermediate values, rendering those for inefficient or extremely efficient enzymes scarce (Figure 4b). This inevitably affects the ability of ML models to generalize to extreme cases. To circumvent this issue, large amounts of evenly distributed high-quality data can be obtained by automating assays through high-throughput systems like biofoundry^155,156^ and microfluidic platforms.^133,157–159^ Biofoundries provide a self-driven laboratory where an agent designs enzymes and deploys them into a characterization unit for synthesis, expression, and kinetic measurement. Then, the agent’s sequence-activity relationship is updated via learning cycles until it converges to an enzyme that meets the query requirements.^155^ Microfluidic platforms can complement this effort by miniaturizing and multiplexing reactions, allowing parallel measurements of kinetic parameters for thousands of enzyme–substrate combinations. Together, these approaches have the potential to generate large, high-quality kinetic data with minimal lab-to-lab variation, covering both well-studied and underrepresented enzyme classes, thereby contributing to the improvements in the performance and generalization of ML models. These automated approaches also provide a realistic pathway to practical closed-loop design–build–test–learn (DBTL) cycles for kinetic modeling.^160^ The ML models may be used to propose candidate sequences, mutations, or enzyme–substrate pairs that are predicted to improve kinetic parameters. The design outputs are then executed by automated strain construction^161^ or cell-free synthesis platforms^162^ and can be characterized via microfluidic assays or in biofoundries. Active-learning strategies implemented in self-driven laboratories prioritize experiments that reduce uncertainty in target regions and accelerate convergence to high-performance variants that can be used to retrain and fine-tune the original ML predictors.^155,163^ To make these loops reliable for kinetic parameter learning, several engineering considerations are required such as rigorous uncertainty quantification,^40^ standardized assay metadata to control for environmental conditions,^77^ calibration steps to reconcile cell-free and *in vitro* measurements with *in vivo* behavior,^69,81,164^ and valid stopping criteria to avoid overfitting to assay artifacts.^155,163,165^

**Figure 4:**
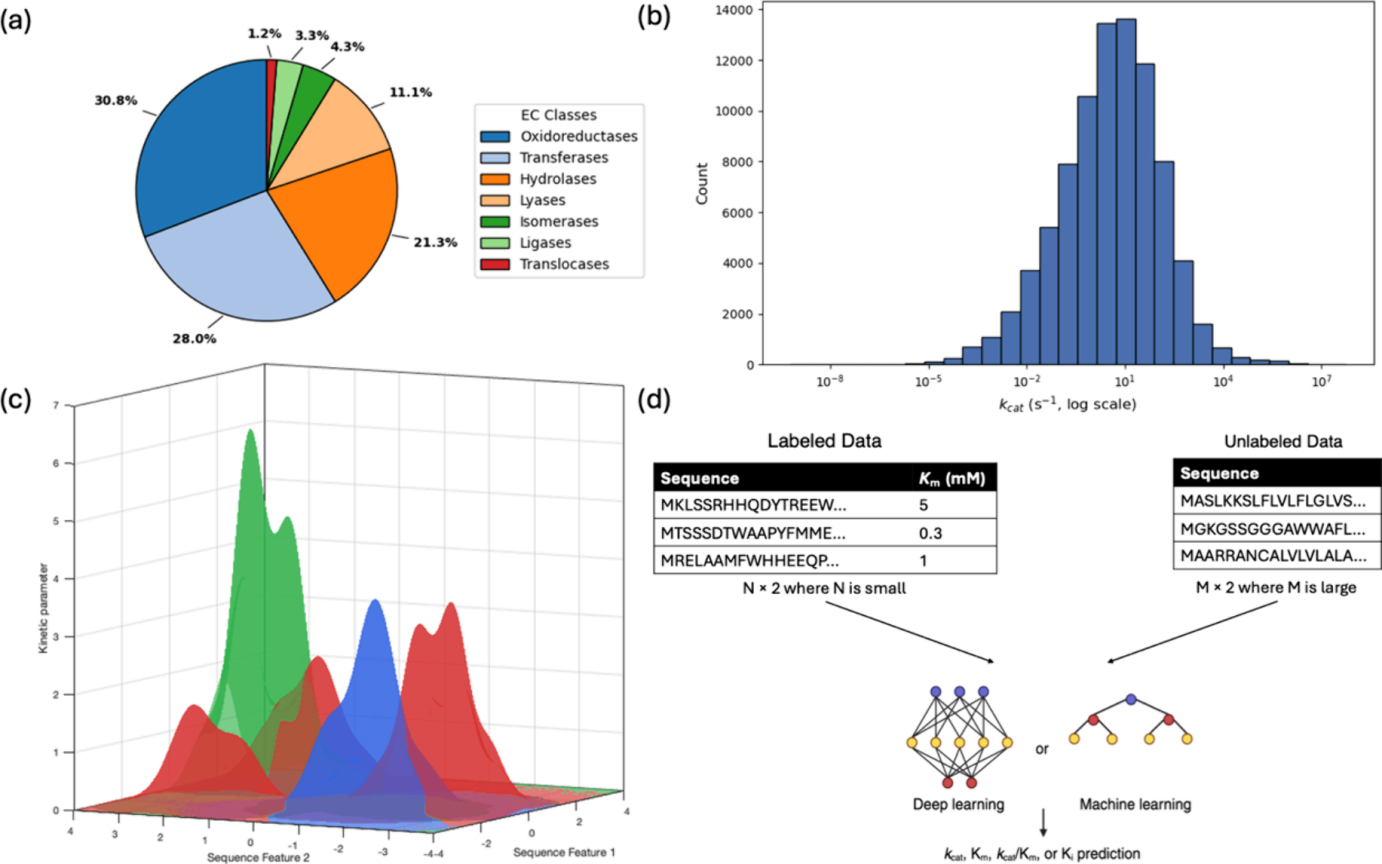
Limitations of current data sets and model types and the use of semisupervised learning to address these limitations. Data imbalance of (a) enzyme classes and (b) *k*_cat_ values in BRENDA. (c) Exploration of functional landscapes using global and local models. Global models are well suited for navigating across diverse enzyme families (represented by different colors), though they provide limited accuracy within the local sequence space (within the same color). In contrast, local models offer more detailed information on a specific functional landscape but do not extend to other enzyme sequence spaces. (d) Schematic of a semisupervised learning approach leveraging labeled and unlabeled enzyme data.

### 7.2 The Need for Hybrid Models

Considering all of the models reviewed here, it is apparent that most of these ML approaches explore either a local or a global view of the sequence space (Figure 4c). This distinction reflects the scope of the search: global models aim to cover a broad and diverse range of enzyme families and classes while local models restrict their focus to specific families. Global models trained on diverse enzyme families generalize across a wide region of sequences. However, they often suffer from low accuracy when predicting parameters for sequences that are highly dissimilar from the training data. Conversely, local models trained on high-quality data sets of wild types or mutants can capture nuanced sequence-function or structure–function relationships. Nevertheless, their predictive power is confined to a narrow region of the sequence space. This is manifested by the difference in performance between the models in Sections 4 and 6, which exemplifies the trade-off between precision and scope.^120^ Accordingly, a promising direction would be to develop hybrid models that leverage global protein language models for a broad contextual depiction of the sequence space while simultaneously fine-tuning on family specific kinetic data sets to retain local information.

### 7.3 Leveraging Semisupervised Learning

Current ML models for kinetic parameter prediction rely almost entirely on supervised learning, which requires large amounts of labeled data for training. Given the limitations discussed above, semisupervised learning offers a promising alternative by leveraging both the limited number of labeled kinetic data and the vast amounts of unlabeled enzymatic sequences in databases like UniProt^59^ (Figure 4d). By exploiting functional patterns in sequence space alongside a limited number of labeled examples, semisupervised frameworks can expand model applicability and improve robustness against data scarcity.^166^

### 7.4 Incorporating Physics-Based Machine Learning

Another promising approach for enzyme kinetic prediction lies in physics-based ML.^167^ Unlike the purely data-driven approaches discussed in this review, physics-based ML embeds biophysical constraints into the learning process to ensure that the predictions remain consistent with the principles of enzyme catalysis. For example, relationships between the activation free energy and *k*_cat_ can be incorporated as constraints during model training. This can be done by regularizing neural networks using penalty terms that enforce consistency with the transition state theory, requiring *k*_cat_ predictions to fall within feasible ranges of activation energies.^168^ Moreover, coupling DL models with quantum mechanics/molecular mechanics (QM/MM) descriptors can assist in capturing molecular mechanisms that govern enzyme kinetics.^169^

Beyond the regression-based predictive models, a recent development is the emergence of generative frameworks that explicitly incorporate biophysical constraints to ensure the mechanistic plausibility of the predicted kinetic parameters. In their 2022 work, Choudhury and colleagues introduced a conditional generative adversarial network that incorporates biophysical and physiochemical constraints to create biologically relevant kinetic models that satisfy thermodynamic requirements, stability constraints, and experimentally observed time scale limits.^170^ Their 2024 work integrates the stoichiometry, regulatory information, flux analysis, and dynamic time scale constraints into the generative process allowing for the estimation of missing kinetic parameters.^171^ Overall, these models outline a blueprint for next-generation ML approaches that integrate statistical learning with the fundamentals of enzyme engineering.

## 8 Conclusion

ML has rapidly emerged as a promising tool for predicting enzyme kinetic parameters. By integrating advances in pLMs and graph-based networks, we are beginning to capture the complex sequence-function relationships that govern catalytic efficiency and enzyme inhibition. These models have the potential to reshape how we explore sequence space, prioritize mutations, and accelerate enzyme discovery. Nevertheless, the field currently suffers from an urgent need for larger and more reliable data sets, which remains the major hurdle to the robustness and generalizability of current predictors. Moving forward, the next generation of models will likely require hybrid strategies that combine the breadth of global pLMs with the precision of family specific fine-tuning. Moreover, incorporating physical constraints and environmental factors will be paramount to bridging the gap between *in silico* predictions and experimental observations. If these challenges are met, then ML will enable the efficient design of enzymes critical in various applications ranging from sustainability to medicine.

## Supplementary Material



## Notes

Relevant definitions and background for machine learning and statistical terms (Tables S1–S3); Additional information on models to predict *k*_cat_ and *K*_m_ (Tables S4 and S5) (PDF)
