# Supplementary material for: Advances in Machine Learning Models for Predicting Enzyme Kinetic Parameters
Source: J Chem Inf Model. 2025 Dec 17;66(1):42–60. doi: 10.1021/acs.jcim.5c02428 (PMC12801326; doi:10.1021/acs.jcim.5c02428)
Supplement: Supplementary file 1 [file ci5c02428_si_001.pdf]

Supporting Information

Advances in Machine Learning Models for Predicting Enzyme Kinetic Parameters

Ali Malli[1], Denys Vasyutyn[1], Jin Ryoun Kim[1,*]

[1] Department of Chemical and Biomolecular Engineering, New York University, 6 MetroTech Center, Brooklyn, NY 11201, United States

* **Corresponding Author:** jin.kim@nyu.edu

Supporting Information – List of Contents

Table S1 – Brief definitions of general terms and concepts used in machine learning

Table S2 – Overview of some types of deep learning network architectures

Table S3 – Summary of some statistical metrics used in the evaluation of machine learning models

Table S4 - Characteristics and performance of additional ML models for predicting k_{cat}

Table S5 - Characteristics and performance of additional ML models for predicting K_m

Table S1 – Brief definitions of general terms and concepts used in machine learning

Term	Abbreviation/Symbol	Definition
Machine learning	ML	General concept of algorithms that learn patterns from data
Deep learning	DL	Machine learning using multi-layer neural networks
Training set	-	The dataset used for model learning
Test set	-	Dataset used to evaluate a trained model
Feature	-	Measurable property used as input to a model
Feature importance	-	Measure of how much each input affects predictions
Embedding	-	Numerical representation of complex data like sequences or molecules
Concatenation	-	Joining multiple vectors into one
Attention mechanism	-	Model component that weights input parts differently depending on relevance
Gate network	-	Mechanism controlling how much of one input passes into the model.
Ensemble model	-	Combination of multiple models to improve performance
Overfitting	-	When a model memorizes training data instead of generalizing
Principal component analysis	PCA	Dimensionality reduction method
Probabilistic regression	-	Regression that outputs a probability distribution, not just a single value
Gaussian distribution	-	Bell-shaped probability distribution
Bayesian multilevel model	BMM	Statistical model estimating parameters at multiple related levels
Synthetic minority oversampling technique	SMOTE	Method to handle class imbalance by creating artificial samples
Data leakage	-	When information from the test set inadvertently appears in training data, inflating performance
Data imbalance	-	Unequal representation of different classes or ranges in the dataset
Cross-validation	-	Method of splitting data into multiple train/test folds to evaluate models
One-hot encoding	-	Method of representing categorical data as binary vectors
Expert layer	-	Specialized parts of a multitask neural network for handling specific subtasks
Multitask learning	-	Training a single model to solve multiple related prediction tasks at once

Pooling	-	Aggregating information from multiple features of a neural network for a more compact representation
Fine-tuning	-	Adjusting a pretrained model's parameters on a smaller task-specific dataset
Transfer learning	-	Reusing knowledge from one task to improve performance on another related task
Diffusion model	-	A generative model that creates candidates while being steered toward a target value predicted by a regression model
Semi-supervised learning	SSL	A machine learning approach that uses a combination of labeled and unlabeled data to train models
Ablation studies	-	Experiments where components of a model are removed to assess their contribution to overall performance

Table S2 – Overview of some types of deep learning network architectures

Term	Abbreviation/Symbol	Definition
Convolutional neural network	CNN	Network for extracting local patterns, often from sequences or images
Graph neural network	GNN	Model operating on graph-structured data
Graph attention network	GAN	Another type of graph-based neural network, uses attention mechanisms
Graph convolutional network	GCN	Graph neural network using convolution-like operations on graph data
Message passing neural network	MPNN	Graph model that passes information along edges and between nodes
Transformer network	-	Architecture using self-attention, popular in NLP and sequence tasks
Pretrained language model	pLM	Model trained on large datasets to learn general patterns in sequences

Table S3 – Summary of some statistical metrics used in the evaluation of machine learning models

**Term**	**Abbreviation/Symbol**	**Definition**
Coefficient of determination	R^2	Measure of how well predictions match actual values
Root mean square error	RMSE	Measure of prediction error size
Pearson's correlation coefficient	PCC, r	Measure of linear correlation between two variables
Mean relative prediction error	-	Average ratio of the prediction error to the true value
Spearman's rank correlation coefficient	r	Statistical measure that assesses the monotonic relationship between two ranked variables
Badness-of-fit	BOF	Measure of how poorly a model explains the observed data, with higher values indicating greater deviation between predicted and experimental results
Enrichment Factor	EF	Measure of how much more frequently top-performing enzymes appear in the model's top-ranked predictions compared to random selection
Precision@*k*	-	Fraction of true top-performing enzymes contained within the model's top *k* ranked predictions
Recall@*k*	-	Fraction of all true top-performing enzymes in the dataset that are successfully captured within the model's top *k* ranked predictions

Table S4 - Characteristics and performance of additional ML models for predicting k_{cat}

Model Name	Dataset size	Model Architecture	Enzyme sequence representation	Substrate representation	Other features	Performance*
NNKcat[1]	16,618	Bagged decision tree ensemble	Long short-term memory model	Attentive fingerprint model	-	R^2 = 0.54
EITLEM-Kinetics[2]	34,429	Neural network	ESM1b	MACCS Keys (RDKit)	-	R^2 = 0.67†
MPEK[3]	17,893	Neural network	ProtT5-XL	Mole-BERT	pH, temperature, organism name	R^2 = 0.65
MMKcat[4]	26,713	Neural network	ESM-2	Pretrained SMILES transformer	Enzyme structure (kNN)	R^2 = 0.57
DEKP[5]	13,401	ExtraTrees	ProtT5-XL-UniRef50	Pretrained SMILES transformer	Enzyme structure (graph transformer), dihedral angles, active site accessible surface area	R^2 = 0.58

*Metrics without a superscript are measured after random data split for training and testing; Metrics labeled with † are measured after sequence-aware data splits for training and testing. R^2 is shown as a primary metric to compare the performance of models.

Table S5 - Characteristics and performance of additional ML models for predicting K_m

Model Name	Dataset size	Model Architecture	Enzyme sequence representation	Substrate representation	Other features	Performance*
PreTKcat[6]	11,722	ExtraTrees	ProtT5-XL	Molecular graph (MolGNet)	Temperature	$R^2 = 0.53$
CataPro[7]	42,018	Neural network	ProtT5-XL	MolT5 and MACCS keys	-	r = 0.63†
SAKPE[8]	53,310	Gradient boosting	ESM-C	Mole-BERT	Catalytic and substrate binding sites from EasIFA	$R^2 = 0.49$†
DEKP[5]	19,847	ExtraTrees	ProtT5-XL	Pretrained SMILES transformer	Enzyme structure (graph transformer), dihedral angles, active site accessible surface area	$R^2 = 0.65$
ENKIE[9]	69,320	Bayesian multilevel models	-	-	MetaNetX reaction identifier, MetaNetX substrate identifier, EC number, enzyme family	$R^2 = 0.46$†
EITLEM-Kinetics[2]	28,664	Neural network	ESM1v	MACCS Keys (RDKit)	-	$R^2 = 0.63$
KinForm[10]	11,722	ExtraTrees	ESMC, ESM-2, ProtT5-XL	Pretrained SMILES transformer	-	$R^2 = 0.53$
ProSmith[11]	11,676	Multimodal Transformer Network	ESM-1b	ChemBERTa2	-	$R^2 = 0.56$†
OmniESI[12]	41,174	Neural network	ESM-2	Molecular graph (GCN)	-	$R^2 = 0.54$†
MMISA-KM[13]	24,585	Neural network	CNNNet	Molecular graph (GCN)	Enzyme structure (GNN)	$R^2 = 0.62$
CPI-Pred[14]	22,588	Neural network	ESM-2	Molecular fingerprint (MPNN)	-	r = 0.65†
RealKcat[15]	44,615	Gradient boosting	ESM-2	ChemBERTa	-	Accuracy = 0.85

*Metrics without a superscript are measured after random data split for training and testing; Metrics labeled with † are measured after sequence-aware data splits for training and testing. R^2 is shown

as a primary metric to compare the performance of models. When R^2 values were not reported, PCC or accuracy were used instead.

References

1. Zhai, J. *et al.* NNKcat: deep neural network to predict catalytic constants (Kcat) by integrating protein sequence and substrate structure with enhanced data imbalance handling. *Brief. Bioinform.* **26**, bbaf212 (2025).
2. Shen, X. *et al.* EITLEM-Kinetics: A deep-learning framework for kinetic parameter prediction of mutant enzymes. *Chem Catal.* **4**, 101094 (2024).
3. Wang, J. *et al.* MPEK: a multitask deep learning framework based on pretrained language models for enzymatic reaction kinetic parameters prediction. *Brief. Bioinform.* **25**, bbae387 (2024).
4. Sun, X., Wang, Y. G. & Shen, Y. A multimodal deep learning framework for enzyme turnover prediction with missing modality. *Comput. Biol. Med.* **193**, 110348 (2025).
5. Wang, Y., Cheng, L., Zhang, Y., Cao, Y. & Alghazzawi, D. DEKP: a deep learning model for enzyme kinetic parameter prediction based on pretrained models and graph neural networks. *Brief. Bioinform.* **26**, bbaf187 (2025).
6. Cai, Y. *et al.* PreTKcat: A pre-trained representation learning and machine learning framework for predicting enzyme turnover number. *Comput. Biol. Chem.* **115**, 108327 (2025).
7. Wang, Z. *et al.* Robust enzyme discovery and engineering with deep learning using CataPro. *Nat. Commun.* **16**, 2736 (2025).
8. Qiu, J.-H. *et al.* SAKPE: A Site Attention Kinetic Parameters Prediction Method for Enzyme Engineering. Preprint at https://doi.org/10.1101/2025.04.30.651216 (2025).
9. Gollub, M. G., Backes, T., Kaltenbach, H.-M. & Stelling, J. ENKIE: a package for predicting enzyme kinetic parameter values and their uncertainties. *Bioinformatics* **40**, btae652 (2024).

10. Alwer, S. & Fleming, R. KinForm: Kinetics Informed Feature Optimised Representation Models for Enzyme k_{cat} and K_{M} Prediction. Preprint at https://doi.org/10.48550/arXiv.2507.14639 (2025).
11. Kroll, A., Ranjan, S. & Lercher, M. J. A multimodal Transformer Network for protein-small molecule interactions enhances predictions of kinase inhibition and enzyme-substrate relationships. *PLOS Comput. Biol.* **20**, e1012100 (2024).
12. Nie, Z. *et al.* OmniESI: A unified framework for enzyme-substrate interaction prediction with progressive conditional deep learning. Preprint at https://doi.org/10.48550/arXiv.2506.17963 (2025).
13. Song, A. & Wang, K. MMISA-KM: a deep-learning method using multi-modal information and self-attention mechanisms for the prediction of Michaelis constants. in *2025 IEEE 14th Data Driven Control and Learning Systems (DDCLS)* 2023–2028 (2025). doi:10.1109/DDCLS66240.2025.11064981.
14. Xu, Z. *et al.* CPI-Pred: A deep learning framework for predicting functional parameters of compound-protein interactions. Preprint at https://doi.org/10.1101/2025.01.16.633372 (2025).
15. Sajeevan, K. A. *et al.* Robust Prediction of Enzyme Variant Kinetics with RealKcat. Preprint at https://doi.org/10.1101/2025.02.10.637555 (2025).
